# Complement C5b-9 and Cancer: Mechanisms of Cell Damage, Cancer Counteractions, and Approaches for Intervention

**DOI:** 10.3389/fimmu.2019.00752

**Published:** 2019-04-10

**Authors:** Zvi Fishelson, Michael Kirschfink

**Affiliations:** ^1^Department of Cell and Developmental Biology, Sackler Faculty of Medicine, Tel Aviv University, Tel Aviv, Israel; ^2^Institute of Immunology, University of Heidelberg, Heidelberg, Germany

**Keywords:** complement, C5b-9, complement-dependent cytotoxicity, regulated necrosis, cancer immune resistance

## Abstract

The interactions of cancer cells with components of the complement system are highly complex, leading to an outcome that is either favorable or detrimental to cancer cells. Currently, we perceive only the “tip of the iceberg” of these interactions. In this review, we focus on the complement terminal C5b-9 complex, known also as the complement membrane attack complex (MAC) and discuss the complexity of its interaction with cancer cells, starting with a discussion of its proposed mode of action in mediating cell death, and continuing with a portrayal of the strategies of evasion exhibited by cancer cells, and closing with a proposal of treatment approaches targeted at evasion strategies. Upon intense complement activation and membrane insertion of sufficient C5b-9 complexes, the afflicted cells undergo regulated necrotic cell death with characteristic damage to intracellular organelles, including mitochondria, and perforation of the plasma membrane. Several pro-lytic factors have been proposed, including elevated intracellular calcium ion concentrations and activated JNK, Bid, RIPK1, RIPK3, and MLKL; however, further research is required to fully characterize the effective cell death signals activated by the C5b-9 complexes. Cancer cells over-express a multitude of protective measures which either block complement activation, thus reducing the number of membrane-inserted C5b-9 complexes, or facilitate the elimination of C5b-9 from the cell surface. Concomitantly, cancer cells activate several protective pathways that counteract the death signals. Blockage of complement activation is mediated by the complement membrane regulatory proteins CD46, CD55, and CD59 and by soluble complement regulators, by proteases that cleave complement proteins and by protein kinases, like CK2, which phosphorylate complement proteins. C5b-9 elimination and inhibition of cell death signals are mediated by caveolin and dynamin, by Hsp70 and Hsp90, by the mitochondrial stress protein mortalin, and by the protein kinases PKC and ERK. It is conceivable that various cancers and cancers at different stages of development will utilize distinct patterns of these and other MAC resistance strategies. In order to enhance the impact of antibody-based therapy on cancer, novel precise reagents that block the most effective protective strategies will have to be designed and applied as adjuvants to the therapeutic antibodies.

## Preface

**Figure 1:**
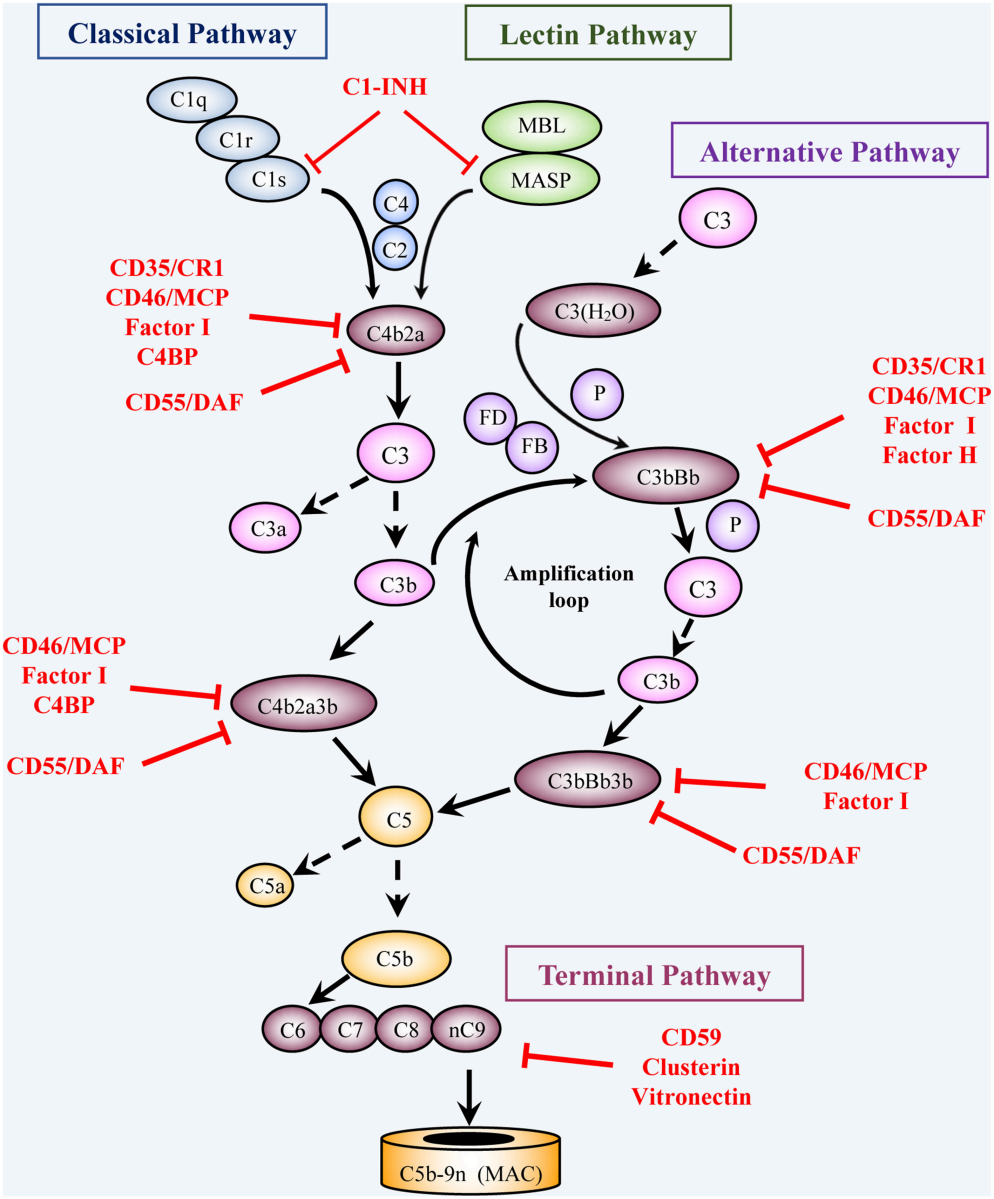
Activation and regulation of the complement pathways. Activation: Complement activation proceeds through four converging pathways shown in this simplified scheme, i.e., the classical (CP), lectin (LP), alternative (AP), and terminal (TP) pathways. Activation of the CP and LP can be potentiated by components of the AP (Amplification loop). Binding of C1 (a complex of C1q, 2C1r, 2C1s) via C1q to antigen-bound antibodies initiates the CP, whereas binding of MBL or ficolin (in complex with MBL-associated serine proteases, MASP) to carbohydrates (e.g., microbial) initiates activation of the LP. The AP is initiated by C3 spontaneously hydrolyzed at a low rate into C3(H_2_O) or following another C3-tickover event. The three pathways generate a C3 converting enzyme, a C3 convertase (that cleaves C3 into C3a and C3b), by activation of C4 and C2 (CP and LP: C4b2a), or of factors B and D (AP: C3bBb). AP activation is facilitated by properdin (P). The resulting C3b not only opsonizes target cells but also joins the C3 convertases and turns them into C5 convertases, which convert C5 into C5a and C5b. Subsequent TP activation by assembly of C5b with C6, C7, C8 and multiple C9 molecules, generates the membrane attack complex, (C5b-9, MAC). By binding to specific receptors, C3a and C5a exert multiple cell stimulatory activities, ranging from allergy and anaphylaxis to promotion of acquired immunity by stimulation of lymphocytes and antigen presenting cells. Regulation: Complement activation is tightly regulated by multiple soluble and membrane proteins. Soluble inhibitors include: C1 inhibitor (C1-INH), C4 binding protein (C4BP), factor H (FH), factor I (FI), Clusterin and Vitronectin. The membrane regulatory proteins are: Decay Accelerating Factor (DAF, CD55), Membrane Cofactor Protein (MCP, CD46), Complement Receptor 1 (CR1, CD35), and CD59. As shown in the figure, C1-INH interferes with activation of C1r, C1s, and MASP. C4BP, FH, CD55, and CD35 restrict formation and stability of the CP and AP C3/C5 convertases or promote FI-mediated inactivation of C4b (CD35/CR1, CD46/MCP, C4BP) or C3b (CD35/CR1, CD46/MCP, FH). Clusterin and vitronectin prevent the association of the forming C5b-9 complexes with the membrane, whereas CD59 limits cell damage by preventing MAC complex formation.

The complement system may affect cancer in several forms, ranging from promotion of cancer growth and metastasis, on the one hand, to antibody-based cancer eradication, on the other. Upon encounter of the cancer cells with the complement system, activation may proceed via the classical, alternative, and/or lectin pathways (1) (Figure 1). This initiation step leads to formation of a C3 convertase (C4b2a or C3bBb) that deposits C3b molecules on the cells, followed by formation of a C5 convertase (C4b2a3b or C3bBb3b) that cleaves C5 and initiates formation of the C5b-9 complexes, termed the membrane attack complexes (MAC). Here, we will focus on the anti-cancer cytotoxic activity of complement, with an emphasis on the mode of action of the MAC. Reviews on the cancer-promoting activities of complement (2–4) and on complement activation by clinical anti-cancer antibodies (5–7) have been published recently; therefore, these topics will not be covered in this review. Another topic recently reviewed is the insights into the fine structural details of the complement MAC (8–11). MAC expresses a plethora of non-lytic and sublytic activities that have been reviewed elsewhere (12–15) and are thus excluded from this review. Here we will describe the current status of research on the cytotoxic effects of MAC, emphasizing the findings, dogmas, and open questions in our quest to better understand the fine mechanistic details of MAC-induced cancer cell death. Next, we will present the currently recognized counter-mechanisms utilized by cancer cells to resist complement-dependent cytotoxicity (CDC). Finally, we will discuss several potential therapeutic approaches for the intervention and potentiation of antibody-based anti-cancer immunotherapy that have been proposed and tested.

## Mechanisms Underlying Complement-mediated Cancer Cell Damage

### Perspective: The Early Studies on Osmotic Cell Death

Studies on cancer cell killing by complement have been conducted long before the identification of the complement terminal pathway responsible for mediating cell damage and death. As early as 1950s, Kalfayan et al. (16), Ellem (17), and Green et al. (18) investigated the action of antibody and complement on rabbit Brown-Pearce carcinoma cells, rat Ehrlich and mouse Krebs ascites tumor cells, respectively. They observed cell swelling and increased plasma membrane leakiness. They proposed that complement impairs cell membrane integrity, increases cell permeability to anions, cations, and water, and causes osmotic cell swelling up to the point that the membrane collapses, culminating in osmotic cell lysis (19). The leakage from the cells was proposed to occur through functional, stretching, and possibly reversible “holes” in the swelling cells, which could be blocked, to some extent, by increasing the osmotic pressure of the extracellular medium (20). The concept of complement-induced osmotic lysis of target cells is still popular today but, as discussed later, it must be viewed with a grain of salt. Kim et al. (21) subjected Ehrlich ascites tumor cells to CDC and demonstrated that osmotic protection effectively prevented cell swelling but did not rescue the cells from death. They hypothesized that the cells died following activation of metabolic events that were detrimental to cell survival or through activation of a “suicidal” mechanism of programmed cell death. In conclusion, osmotic burst of inflated complement-damaged cells may occur, but these bursts are most likely a consequence of metabolic collapse of the cell rather than the cause of cell death.

### The Complement Cell Death Mediator: A Concerted Action of Toxic Moieties

Membrane pores caused by complement were first visualized by electron microscopy on red blood cell membranes as large ring structures (22). Similar lesions were viewed on *E. coli* cell walls (23). Over the years, ample information on the fine ultrastructure of the MAC that can activate cell death has been gathered (24) and has been recently further examined (8–11, 25–27). For a complete updated view of the MAC structure, the reader is referred to those publications. The observed ring structure apparently corresponds to the structure of polymerized C9 molecules attached to their polymerization accelerator, the C5b-8 complex (28). However, even today we have only a partial view of the fine details of the cytotoxic mechanisms activated by MAC, eventually leading to the point of no return and cell death. Besides the paucity of investigations on the subject, several reasons account for that. First, the early dogmas were based on investigations with complement-targeted artificial membranes and red blood cells, which are clearly different, largely passive targets, compared with nucleated cells (29–34). Second, very large variation exists in refractoriness to the MAC, even among closely related cancer cell lines and even within a supposedly homogenous population of cultured cancer cell lines. Third, in target cells MAC activates concomitantly several signaling pathways and biochemical events, some cytotoxic and others pro-survival, and it is the particular balance among them that dictates cell fate, survival, or death. Finally, activation of the terminal complement pathway may result in generating, in the target cell membrane, a cocktail of membrane-inserted protein complexes: C5b-8, C5b-9_1_, C5b-9_2_, C5b-9_3_, and so on, up to C5b-9 with 12-18 polymerized C9 molecules (28, 35). Each of these complexes may induce in the target cell slightly different signals that have not yet been discretely characterized. Detailed analysis of the effect of the terminal complement complex size on the lysis of rat Ehrlich ascites tumor cells by human complement indicated that complexes containing more C9 per C5b-8 are cytolytically more potent. Nevertheless, the kinetics of cell death appeared similar in cells bearing C5b-9 complexes that have either 1 or 4 C9 molecules per C5b-8 (36). Moreover, some human cancer cells, such as U938, HL60, and B-CLL cells, could be lysed by C5b-8 alone, in the absence of C9, when a sufficient number of complexes were deposited on them (37, 38). Hemolysis of sheep red blood cells could be efficiently activated by C5b-9 complexes generated with thrombin-cleaved C9, which cannot undergo classic ring-like polymerization, but forms apparently, string-like oligomeric structures that may lead to leakage of membranes (39, 40). Hence, it is improbable that MAC, with its various intermediary complexes, activates a unified mechanism of cell death in all cell types. An additional level of complexity has been introduced by reports of apoptotic cell death induced by MAC (41), but this has not been observed so far with cancer cells undergoing CDC.

### Calcium Ions Influx: Dose-Dependent Dichotomy

At non-toxic or sublytic doses, MAC has been shown to trigger numerous signals in many types of cells, normal and malignant. This topic has been extensively discussed recently and will not be covered here (12–15). Initially, measurements with pigeon erythrocyte sealed “ghosts” revealed an increase in intracellular calcium ions, which begins within seconds after binding of MAC and supposedly precedes the cell death process activated by lytic doses of MAC (42). This transient rise of intracellular free Ca^2+^ in target cells was thought to be required for cell death. However, later it became apparent that the rise in the level of intracellular calcium ions is essential for cell survival and recovery (43). Reduction of the extracellular Ca^2+^ concentration by chelation delays the onset of cell death, as measured by LDH release, but the cells eventually die like control cells (44, 45). Similarly, increasing the concentration of Ca^2+^ around the cells accelerates the rate of cell death without affecting the final percentage of dead cells (36). An intriguing question is: can CDC be blocked by intracellular chelation of the calcium ions? Intracellular Ca^2+^ chelation with BAPTA-AM was shown to efficiently block mitochondrial distress in human lung epithelial cells responding to a non-lytic dose of MAC, cells that do not undergo cell death (46). Furthermore, calcium ionophores that pump Ca^2+^ into the cell induce in K562, human erythroleukemia cells, a state of resistance to CDC (47). Can BAPTA-AM block CDC when cells are exposed to lytic MAC doses? BAPTA-AM reduced the release of LDH from rat hepatocytes subjected to lytic antibody and complement by ~40% without affecting the rate of cell death (48). Clearly, MAC activates a surge of [Ca^2+^]_i_ in target cells but its exact impact on the process of cell death still awaits clarification. Furthermore, based on earlier findings, the involvement of calcium-independent processes in the critical events determining cell death cannot be ruled out.

### Beyond Calcium Ions: The Cell Death Propagators in a Regulated Necrotic Process

**Figure 2:**
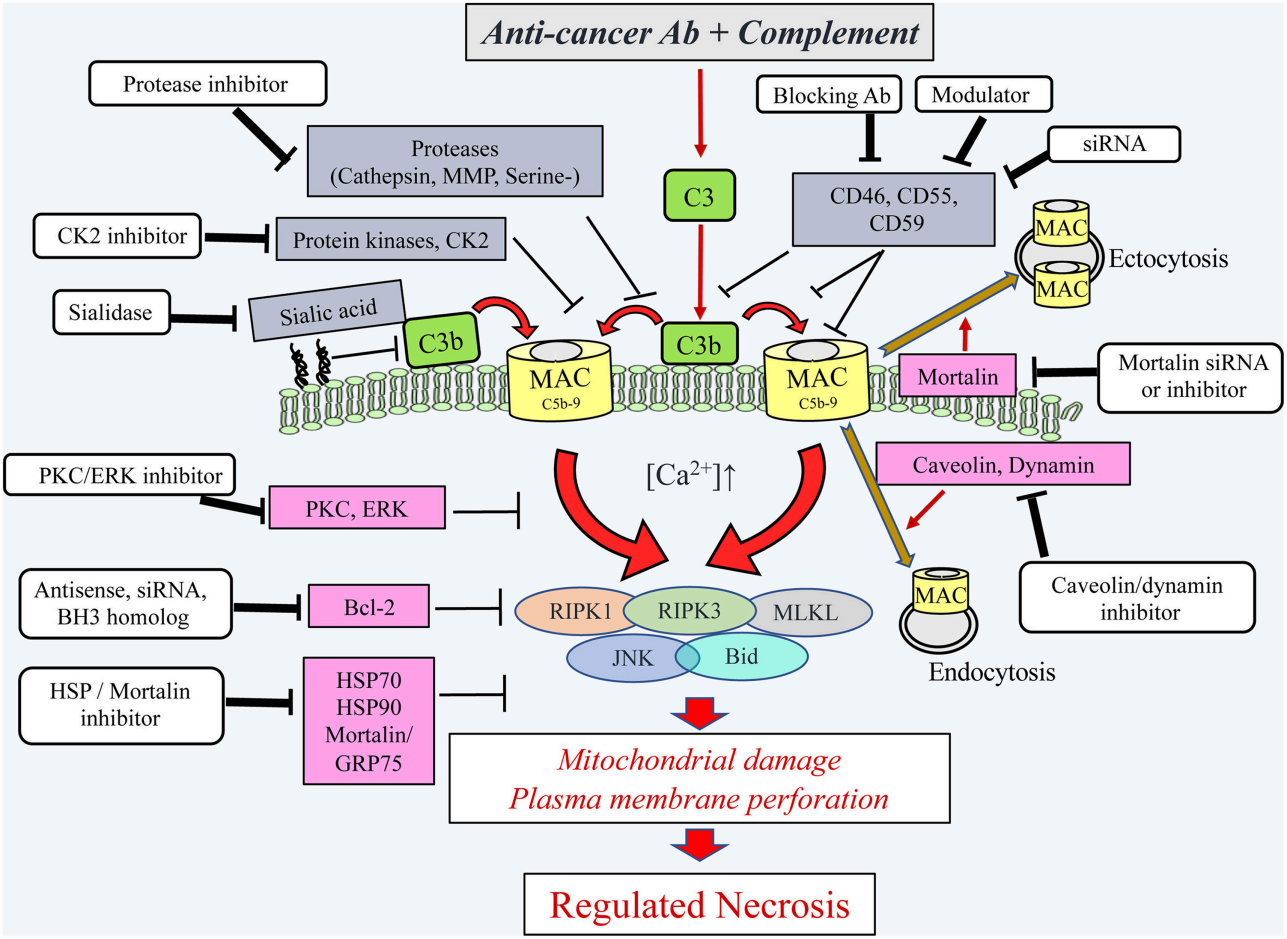
Schematic presentation of the cytotoxic pathways, induced in cancer cells by the complement C5b-9, the counteractive cellular resistance mechanisms, and postulated approaches to overcome this cancer evasion. Following the binding of antibodies to cancer cells, the complement system is activated and deposits C4b and C3b molecules that serve as initiators of C3/C5 convertase activation. The C5 convertases initiate the activation of the terminal complement pathway and the formation of the C5b-9 complexes (24). Upon insertion of the C5b-9 complexes into the plasma membrane of cancer cells, they induce calcium ion influx and activate pro- and anti-lytic signals. This scheme depicts the proteins proposed to be involved in the ensuing cancer cell death (encircled) and the proteins protecting the cancer cells from the lytic processes. Extracellular (gray boxes) and intracellular (purple boxes) protective proteins are indicated. Several reagents (white boxes) that will block the protective proteins are indicated and proposed for adjuvant therapy to therapeutic antibodies. Ab, antibody; Bcl-2, B-cell lymphoma/leukemia-2; BH3, Bcl-2 homolog domain-3; Bid, BH3 interacting domain death agonist; CK2, casein kinase 2; ERK, extracellular signal-regulated kinase; HSP90, heat shock protein 90; HSP70, heat shock protein 70; JNK, c-jun N-terminal kinase; MAC, complement membrane attack complex; MLKL, mixed lineage kinase domain-like protein; MMP, matrix metalloproteinase; PKC, protein kinase C; RIPK1, receptor-interacting protein kinase 1; RIPK3, receptor-interacting protein kinase 3; serine-, serine protease; siRNA, small interfering RNA.

The molecular checkpoints that tilt the balance within MAC-bearing cells between a protective state and cell collapse have not yet been identified. It is well-accepted that exposure of nucleated cells to multiple (“lytic”) MAC hits (34) is needed to overcome the cells' innate resistance (described below) and to kill the cells by necrotic-type cell death. Intensive research on apoptosis, and more recently on necroptosis induced by numerous effector molecules, has clearly revealed that compound regulated molecular processes accompany and/or lead to cell death (49–52). Those findings have prompted adopting a similar research approach in the analysis of the mechanism underlying CDC. Recently, MAC was shown to activate RIPK1, RIPK3, and MLKL, known transducers of necroptotic cell death activated by several exogenous ligands of TNF receptor, Fas, TLR, and other membrane receptors (53). Necroptotic cell death, also termed regulated necrosis, is characterized by increased membrane permeabilization and mitochondrial damage (49, 50, 54, 55), much like CDC. Inhibitors of RIPK1, RIPK3, and MLKL reduce the extent of CDC, whereas overexpression of these proteins enhances cell sensitivity to CDC (53). Two additional intracellular proteins that may play a role in the multi-factorial cell death process activated by lytic MAC are the c-Jun kinase JNK (56) and the BH3-only protein Bid (57). Apparently, in some cells, the RIP kinases, MLKL, JNK, and Bid, act as components in one or more lined cascades of intracellular molecular interactions activated by sublytic and lytic MAC concentrations (53, 57). At lytic MAC concentrations, this cascade may promote a regulated necrotic cell death (Figure 2). Blocking any of these five proteins markedly lowers the extent of CDC but does not block it completely. Therefore, it appears that this cascade acts in concert with other death-promoting processes, calcium-dependent or -independent, which still await characterization. Of note, activated MLKL was shown in necroptotic cells to oligomerize at the plasma membrane, increase membrane permeabilization, and induce a Ca^2+^ influx (55, 58–60). Co-localization of MAC with MLKL at the plasma membrane (53) suggests that they may collaborate in mediating cell death. In general, cancer cells that express sufficient levels of the RIPKs, MLKL, and Bid might be sensitive to this necroptotic-like pathway once activated by MAC. In contrast, cancers that suppress the expression or function of any or all of these proteins are expected to be protected from this cytotoxic pathway even if triggered by MAC.

### Lytic MAC: Mitotoxicity and Metabolic Depletion

Mitochondria play a pivotal active role in activating the intrinsic pathway of apoptotic cell death, mostly after mitochondrial outer membrane permeabilization (61). Mitochondrial swelling and damage were observed in cells undergoing necrotic cell death induced by complement (62, 63). The cellular ATP level drops rapidly in cells attacked by MAC in copy numbers that are above the lytic threshold, apparently after mitochondrial dysfunction, accompanied by leakage of cytosolic ATP from the cells through pores in the plasma membrane (64–66). In theory, mitochondrial damage and cellular metabolic depletion beyond a point of “no return” may induce cell collapse and death. However, to date, there is no strong evidence supporting an active role for mitochondria in CDC. Perhaps, they are mere innocent bystanders damaged by the necrosis executioners? Reactive oxygen species are generated throughout the necrotic process (15). Still, whether they take part in the MAC-induced cell death process is also an open question. The involvement of mitochondria and mitochondrial ROS in necroptosis triggered by various necroptosis inducers was extensively investigated in several types of target cells (67). Ample earlier findings have supported a pivotal role for mitochondria in necroptosis, but more recently, several investigations casted doubt on that notion (67). Thus, for example, mitochondrially deficient cells were shown to be responsive to TNF/zVAD treatment and to undergo necroptosis (68). It will be of interest to examine the relative sensitivity of these cells to CDC. At present, we can conclude that whether mitochondria are dispensable or essential for MAC-induced necrotic cell death remains to be further investigated. Similar to necroptosis (67), it is likely that different cell types may mediate CDC by an array of distinct mitochondria-dependent and -independent strategies.

## Normal Cells and Even More so, Cancer Cells Can Resist Mac-induced Cell Death

The fate of a target cell attacked by MAC is dictated by two mutually exclusive processes: (a) the rate and extent of the formation of the C5b-9 complexes and their insertion into the target cell membrane, and (b) the capacity of the target cell to block C5b-9 complex formation and to resist cell damage inflicted by C5b-9. Cancer cells can resist CDC by using a plethora of extracellular and intracellular mechanisms (Figure 2). The number of membrane-inserted C5b-9 complexes may be restricted by inhibiting the complement activation cascade earlier at the C3/C5 activation stage, by blocking complex assembly and/or membrane insertion, and by facilitating complex removal from the cell surface. All these protective strategies have been identified in cancer cells and are described below. It is generally accepted that cancer cells are more resistant to CDC than are normal cells due to their elevated expression of protective mechanisms. Apparently, during the tumorigenic process, the complement system reacts against the transformed cells by eliminating or modifying the complement-sensitive malignant cells, thus enriching the cancer cell population for complement-resistant cells. This process resembles the selection of antibiotic-resistant bacteria (69). This hypothesis remains to be supported by *in vivo* evidence; however, *in vitro* studies show that sensitive cancer cells may be transformed into cells expressing increased complement resistance, transiently, after a brief treatment with a sublytic dose of MAC (70) and more stably, following several cycles of exposures to cytolytic MAC (71). A MAC-resistant phenotype may be acquired upon the elevation or reduction in the expression level of microRNAs such as miR-200, miR-217 (72), and others that are currently under investigation.

### Basal Physiological Cell Resistance to CDC

As shown already in 1974 (73, 74), nucleated cells can resist MAC-induced damage. Several inhibitors of protein synthesis were shown to increase the cell's susceptibility to CDC. Since the elevated sensitivity to CDC was acquired hours after the complete shutdown of protein synthesis (75), it is likely that the treated cells became sensitive only after catabolism of long-lived protective proteins. These earlier findings were followed by the development of the concept of multi-hit characteristics of nucleated cell death by MAC, implicating the cooperation of multiple MACs in cell death (34). Another interesting earlier finding was that damaged tumor and mast cells could be rescued by exogenous application of cAMP (76, 77). Consequently, activation of cAMP by sublytic MAC supports cell recovery from MAC damage (78). These findings were confirmed in leukemia cells treated with dibutyryl cAMP or with activators of intracellular cAMP (3-isobutyl 1-methyl xanthine and forskolin), which were shown to reduce cell death (79). In contrast, H-89, an inhibitor of the cAMP-dependent kinase PKA, enhanced carcinoma cell sensitivity to CDC. Apparently, phosphorylation events mediated by several protein kinases dictate the basal capacity of cells to resist MAC damage (79, 80). Protein phosphorylation events involving PKC, MEK, and ERK support the survival of cancer cells undergoing a complement attack (81–83). Protein phosphorylation may upregulate the expression of the complement membrane regulators on cancer cells (84–86) and facilitate MAC elimination from K562 cells (87, 88). The transcription factor NF-κB also plays a role in cell protection from CDC (89). One of its postulated functions is upregulation of a protein phosphatase that inactivates JNK, thus reducing cell death signaling. However, further investigation is required to fully identify the pro-survival phosphoproteins and phosphatases and their precise mode of action.

Proteins of the heat shock protein family (HSPs), well-known general house keepers, damage/ repair proteins and targets in cancer therapy (90–92), most probably also contribute to the basal resistance of cancer cells to CDC. Thus, far, a role for Hsc70/Hsp70 (93) and Hsp90 (94, 95) in cell protection from CDC has been shown. Pharmaceutical inhibitors of Hsp70 and Hsp90 sensitize cancer cells to CDC. Hsc70 relocates within minutes from the cytoplasm to the cell surface after exposing K562 cells to sublytic complement (93). Upon inhibition of Hsp90, Ramos cells become more sensitive to the action of Rituximab and complement (95). Additional thorough experimentation is required to fully comprehend how Hsc70/Hsp70, Hsp90, and other HSPs regulate CDC. The fact that Hsp90 can directly interact with C9 and that Hsp90 inhibitor enhances MAC deposition (95) suggest that Hsp90 down-regulates MAC deposition by blocking its assembly and/or facilitating its rate of removal from the cell surface. Hsp90 can potentially reduce CDC by suppressing mitochondria-initiated calcium-mediated stress responses (96).

### Anticomplementary Response on the Cancer Cell Surface

Like all normal cells, cancer cells are protected from autologous complement attack by several specific cell-surface complement inhibitors: CD55 (decay accelerating factor, DAF), CD46 (membrane cofactor protein, MCP), CD59, and CD35 (complement receptor type 1, CR1) (1, 97–99) (Figure 1). In addition, certain proteolytic enzymes, protein kinases, and sialic acid residues (described below) confer on the cells elevated resistance to CDC (100).

#### Membrane Complement Regulatory Proteins

Immunohistochemical analysis revealed the expression of CD59, CD55, and CD46 on uveal melanoma (101), thyroid carcinoma (102), lung and kidney cancer (103, 104), colon adenocarcinoma (105), and prostate cancer (106). This was supported by analysis of human tumor cell lines derived from human malignant gastrointestinal tumors (107), melanoma (108), breast cancer (109, 110), renal tumor (110), Burkitt lymphoma (111), neuroblastoma (112), and ovarian (113), and prostate carcinoma (79). In primary uterine cervix tissue, the expression of CD46, but not of CD55, was found to increase during transition from normal to premalignant to malignant cells (114). CR1/CD35 was identified in malignant endometrial tissue (115) and on leukemic blasts (116). Increased membrane regulator expression relative to the corresponding normal tissue has been reported in many tumors (103, 105, 114, 115, 117–122). Colorectal and gastric carcinomas and osteosarcoma have increased the expression of CD55 (123), whereas gastric carcinoma exhibits high levels of both CD55 and CD59 (124). Overexpression of CD59 was also identified by expression profiling for pancreatic cancer (125). Upregulation of membrane regulator expression on tumor cells is often correlated with increased complement resistance (86, 111). In ovarian cancer, resistance to complement correlated with high levels of CD55 expression (113). In melanoma cell lines with variable CD59 expression, resistance to death by anti-ganglioside antibody and homologous complement positively correlated with the expression level of CD59 (126).

Several clinical studies support a postulated function of membrane complement regulatory proteins (and thus, the extent of complement resistance) in cancer progression. Poorer prognosis in colorectal carcinoma correlates with the expression level of CD59 (127). Local tumor progression and tissue dedifferentiation of prostate cancer also correlate with CD59 expression (128). Analysis of 120 breast cancer patients revealed a worse prognosis associated with CD59 overexpression (129). In contrast, another report concluded that loss of CD59 correlated with poor survival in 520 breast cancer patients (130). In colorectal cancer patients, a 7-year survival was significantly reduced when the tumors expressed high levels of CD55 (131). CD55 overexpression was also reported as an independent risk factor for recurrence of breast cancer in patients receiving postoperative adjuvant therapy containing trastuzumab (132).

The expression level of the membrane complement regulators may also be shaped by cytokines, growth factors, or hormones, which are released into the tumor microenvironment (83, 133, 134). For example, TNFα and IL-1ß enhanced the expression of CD55 and CD59 in colon adenocarcinoma cells (135). TNFα, IL-1α, and INFγ enhanced CD55 expression in lung cancer cells (136). In hepatoma cells, TNFα, combined with IL-1ß and IL-6, enhanced CD55 and CD59 expression but decreased CD46 expression (134). Transcription abnormalities (137) and the microRNA level of expression (72) may also affect the expression level of the membrane complement regulators. Evidently, the factors and molecular mechanisms that determine the expression level of each of the membrane regulator proteins *in vivo* in each cancer type (and in normal cells) remain to be further investigated.

Exposure to chemotherapeutic drugs may also modify the level of the regulators′ expression. 5-azacytidine was shown to elevate the levels of CD55 and CD59 in Burkitt lymphoma cell lines (111) but only of CD59 in melanoma cells. In contrast, levamisole reduces CD59 levels in colon adenocarcinoma cell lines (138) and after pretreatment of breast carcinoma cells with tamoxifen, trastuzumab-induced CDC was enhanced due to CD55 down-regulation (132). Conversion of cancer cells from being drug-sensitive to drug-resistant is also associated with modification of their complement sensitivity. Doxorubicin-resistant human colon carcinoma cells are more sensitive to CDC than are doxorubicin-sensitive cells (139). KB-V1, a multidrug-resistant variant of KB-3-1, the human oral carcinoma cell line, exhibits a higher susceptibility to CDC than do its parental multidrug-sensitive cells (140). The increased complement sensitivity was associated with a reduced expression of CD55. Inversely, drug resistance was associated with CDC resistance in the HL60 myeloid leukemia cell lines (141). In ovarian carcinoma cells, drug resistance was associated with complement resistance and with membrane complement regulator overexpression (142). Hence, the impact of any drug on the expression of membrane regulators and on CDC resistance needs to be determined for each drug and cancer type.

Released or secreted membrane complement regulators in the cancer microenvironment may also support cell resistance to CDC. Soluble forms of membrane regulators have been identified in several body fluids, even under normal conditions. They are either produced by alternative splicing or released from the cell surface through enzymatic cleavage. Thus, sera of cancer patients contain active, soluble forms of CD46 (143). Elevated CD55 concentrations in stool specimens have been proposed to have diagnostic value for patients with colorectal cancer (144). A constitutive release of soluble CD59, which retains its activity as well as its GPI-anchor from human melanoma cells, was reported (145). In primary tumor sections, CD55 and/or CD59 were found in the stroma of breast, colorectal, lung, renal, and cervical carcinomas (103, 123, 146). *In vitro*, endothelial cells, HeLa cells (147) as well as osteosarcoma and colorectal cells (123, 148) release CD55 in a soluble form or deposit it into their extracellular matrix. K562 erythroleukemia cells (83) and breast, ovarian, and prostate carcinoma cell lines (79) secrete soluble CD59. Elevated plasma levels of soluble CR1 were found in leukemia patients (149).

The observed correlations between elevated expression or secretion of one or more of the membrane complement regulatory proteins on cancer cells and (a) enhanced resistance to CDC or (b) poor cancer prognosis, suggests that the membrane complement regulatory proteins have an effect on prognosis through their impact on complement resistance. Thus, by suppressing C3 deposition on the cancer cells, CD46 and CD55 can lower, on one hand, the extent of MAC generation and CDC, and on the other hand, reduce immune protection through complement-dependent cellular cytotoxicity. CD59 can down-regulate MAC generation and CDC. However, a direct correlation between cancer patients' prognosis and the complement resistance level of their cancer, still remains to be established. We cannot rule out non-complement-mediated effects of the membrane complement regulatory proteins of cancer cells on the patients' immune response. Thus, the membrane complement regulatory proteins on cancer cells, through intracellular signaling, or cooperation with other cell surface receptors may potentially modulate cell resistance to immune effector cells such as natural killer cells and cytotoxic T lymphocytes (150, 151).

#### Membrane Surface Proteases, Protein Kinases, and Sialic Acid

Cancer cells become increasingly protected from CDC by expression on their cell surface of proteases that proteolytically degrade the deposited complement proteins (152). Thus, degradation of bound C3b by a C3-cleaving serine (153) or cysteine protease (154), respectively, was demonstrated on human and murine melanoma cells. C3-cleaving serine protease activity was also identified on the surface of U937 cells (155). Membrane serine proteases on K562 erythroleukemia cells also appear to contribute to their complement resistance (156). Matrix metalloproteinases (MMP) membrane type-1 (MT1) can cleave bound C3b off breast cancer cells and protect *in vitro* breast carcinoma and melanoma cells from CDC (157). Transfection of B16F1 melanoma cells with MT1-MMP enhanced their capacity to form lung metastases in normal but not in C3-deficient C57BL/6 mice (157). The effect of those proteases on proteins of the terminal complement pathway has not been tested. However, it is conceivable that these and other membrane proteases have a similar degradative impact on C5-C9. This still awaits determination.

Ecto-protein kinases (ecto-PK) are extracellular protein kinases that can phosphorylate both cell-surface and external proteins. Serine/threonine and tyrosine ecto-PKs were found on the surfaces of K562, U937, and HL-60 cells (158), and an ecto-casein kinase 2 (CK2)-like activity was associated with breast and ovarian carcinoma cells (159). C9 phosphorylation by ecto-CK2 was shown to be protective in K562 cells against CDC, possibly by inhibiting MAC formation or by leading to the production of an inactive or unstable MAC (160). Further investigation of this strategy of CDC evasion is warranted.

Brief treatment with sialidase, which removes sialic acid from the cell surface, has been shown to confer on several cell types increased sensitivity to CDC. Thus, removal of sialic acid from red blood cells (161, 162), murine sarcoma cells (163), and human bladder carcinoma cells (164) sensitized them to lysis by complement. Human prostate, breast, and ovarian carcinoma cells also utilize surface sialylation for protection from complement (79). High sialic acid expression correlates with lower complement activation, probably because of inactivation of C3b by factors H and I, which is more efficient on surfaces rich in sialic acid (162). The sialic acid inhibitory activity on CDC of mouse erythrolukemia MEL cells is apparently abrogated by O-acetylation at its 9-hydroxyl group (165). α2-6 hypersialylation apparently lowers the response of CLL cells to Rituximab therapy through its action on complement (166). Thus, by limiting the extent of C3 deposition, sialic acid may also control the assembly of C5b-9 complexes on the cancer cells.

### Soluble Complement Regulators in the Cancer Microenvironment

Soluble complement inhibitors such as C1 Inhibitor, factor H, and factor I are predominantly synthesized by hepatocytes and macrophages but can also be released from other tissues, although in considerably smaller amounts. In the cancer microenvironment, these secreted inhibitors may contribute to protection of cancer cells from complement attack by blocking complement activation at the C1 and C3 activation steps (99). In support of this, a growing number of reports indicate that cancer cells of various origins secrete one or more complement inhibitor. Synthesis of C1 Inhibitor has been described in astroglioma and neuroblastoma (112), breast cancer cell lines, and in a primary ovarian carcinoma cell line (156). Factor H is expressed both in lung adenocarcinoma and cutaneous squamous cell carcinoma (167, 168) and high levels of factor H and factor H-like protein-1 were shown to be secreted by ovarian tumor cells (83, 169). Additionally, factor H was found to be elevated in bronchoalveolar lavage fluids and the sputum of patients with lung cancer (170). Chronic lymphocytic leukemia (CLL) cells that bind factor H to their surface resisted Rituximab-mediated CDC (171). Factor H was coexpressed with factor I in glioma and rhabdomyosarcoma cells in its plasma form and in a truncated form (172). Tumor-associated factor I is postulated to promote the progression of cutaneous squamous cell carcinoma (173) and positively correlates with poor survival and recurrence of breast cancer (174).

### Active Removal of the Membrane-Inserted MAC

An additional important defensive tactic used by cancer cells to resist CDC is rapid elimination of MAC from the cell surface. This was first shown with U937 histiocytic leukemia cells, Ehrlich ascites tumor cells (175, 176), and neutrophils (177). Neutrophils remove MAC both by endocytosis and exocytosis (178). Elimination of MAC by exo-vesiculation has been described in glomerular epithelial cells, platelets, and oligodendrocytes (179–181). The intracellular signals involved in MAC elimination include Gi proteins (182), PKC and ERK (88, 183). The process of MAC removal through outward and inward vesiculation was imaged in MAC-bearing K562 erythroleukemia cells (184). Membrane vesicles shed from MAC-bearing neutrophils contain MAC and have elevated levels of cholesterol and diacylglycerol, suggesting selective membrane protein and lipid sorting during the ectocytosis process (185). In support, the elimination of MAC by endocytosis is inhibited in K562 cells after cholesterol depletion (186). MAC endocytosis in K562 cells largely depends on caveolae and dynamin-dependent intracellular release of MAC-loaded endosomes (186). The process of MAC removal by exo-vesiculation was also partially characterized in K562 cells and was found to require the expression of the mitochondrial stress protein mortalin/GRP75 (87). Mortalin is over-expressed in many cancer types and is an essential survival stress protein (187). It was shown to be significantly protective from CDC (188). Its exact mode of action remains to be elucidated; however, evidently, mortalin inhibitors efficiently sensitize K562 cells and colorectal carcinoma HCT116 cells to CDC (189).

## Intervention Strategies to Overcome Cancer Resistance to CDC

As previously described, cancer cells escape CDC through amplification of an array of resistance strategies that block the formation of MAC, facilitate MAC elimination from the cell surface, or inhibit the cytotoxic consequences of MAC insertion into the plasma membrane (Figure 2). In order to overcome that resistance, more potent antibodies and polymeric antibodies have been engineered (5–7). Attachment of complement-activating proteins such as CVF, C3b, C7, or C9 directly to therapeutic antibodies represents an alternative means to strengthen complement attack and thereby to overcome complement resistance of cancer cells (190–193). Here, we will restrict our description to intervention strategies that may be or have been developed to augment the CDC of cancer cells by weakening their anti-MAC resistance mechanisms. These include the following: (1) blocking or silencing the membrane complement regulatory proteins, (2) inhibiting the extracellular enzymes that interfere with complement activation, and (3) inhibiting the intracellular pathways that support cell resistance and recovery (Figure 2). An additional, yet unexplored approach, which is based on the earlier findings, is targeting a sialidase to the cancer microenvironment or blocking the sialylation of surface glycoconjugates in cancer cells, which is expected to sensitize them to CDC.

### Antibody-Mediated Neutralization of Complement Regulator Expression

Specific inhibition of complement regulators' activity is best achieved with monoclonal antibodies that enhance the susceptibility of cancer cells to CDC (86, 194, 195). Thus, blocking antibodies markedly enhance the anti-tumor activity of Rituximab *in vitro* and *in vivo* (196). Neutralization of CD55 in Burkitt lymphoma cells (111), leukemia cells (196–199), melanoma cells (200), and breast cancer cells (86) increased their sensitivity to complement. Similarly, inhibition of CD59 with a monoclonal antibody led to efficient sensitization to CDC of neuroblastoma cells (112), leukemic cells (83, 199), breast (86), ovarian (113), renal (201), and prostate carcinoma cells (106). Mini-antibodies targeting both CD55 and CD59 were shown to enhance Rituximab-dependent CDC *in vitro* and to increase the survival of Rituximab-treated SCID mice in a xenograft model of human CD20^+^ B-cell lymphoma (196). Bispecific antibodies targeting both CD20 and CD55 or CD20 and CD59 were also shown to potentiate the CDC of CD20-positive lymphoma cells *in vitro* and to prevent the growth of human lymphoma cells in SCID mice (202). Neutralization of the soluble complement regulators may also be applied to cancer immunotherapy. Thus, anti-factor H antibody increased antibody-dependent CDC of colorectal cancer treated with anti-CEA monoclonal antibody (203). Inhibition of factor H activity with a recombinant protein reflecting the factor H short-consensus repeat 18–20 improved the CDC of CLL cells in the presence of Rituximab and the blockage of CD55 and CD59 further enhanced CDC (171).

### Silencing of Complement Regulators' Expression by RNAi

Another specific approach is to knock down the expression of the membrane complement regulators' expression by siRNAs. RNA interference (RNAi), mediated by small interfering RNA (siRNA), is the most efficient strategy for specific silencing of therapeutically relevant genes (204). In the last years numerous strategies have been developed for a better delivery of siRNAs in *vitro* and *in vivo* (205). We have shown that silencing of single or multiple complement regulators by anti-sense oligonucleotides or siRNAs results in a significant increase of opsonization and CDC of tumor cell lines of various histological origin (194, 206). Silencing of CD55 and CD59 in breast cancer cells with specific shRNA enhanced CDC (207). Using chemically stabilized anti-complement regulators, siRNAs and AtuPLEX, we observed a significant knockdown of regulator expression on HER2-positive carcinoma cells. Subsequently, treatment with a combination of two anti-HER2 antibodies, trastuzumab and pertuzumab, and normal human serum, augmented C3 binding and CDC could be recorded (208). Similar results were observed with lymphoma cells in which silencing of complement regulators enhanced antibody-dependent CDC (209). For specific delivery of liposomes or lipoplexes loaded with siRNA molecules into cancer cells, transferrin may be attached to them to facilitate their binding to cancer cells through transferrin receptor (TfR/CD71) and their active entry into the cells (210). By using this approach, delivery of siRNA molecules specific to CD46, CD55, and CD59 to transferrin receptor-positive carcinoma cells was achieved and promoted the knockdown of the complement regulators and enhanced CDC (211).

### Neutralization of Extracellular Protective Enzymes

Considering the aforementioned anti-complement effects of certain proteases and protein kinases, it is likely that tailor-made protease and/or kinase inhibitors will promote antibody-based immunotherapy. *In vitro* and a few *in vivo* results support this hypothesis. Treatment of K562 cells with serine protease inhibitors markedly enhanced their sensitivity to CDC (156). CK2 inhibitors also augmented Raji cell killing by Rituximab and complement (160). Single-chain variable fragment (ScFv) directed to cathepsin L was used to inhibit the tumorigenic and metastatic phenotype of human melanoma cells in nude mice (212). In addition, injection of an anti-cathepsin L ScFv lentiviral vector into tumors already induced in nude mice inhibited tumor growth and associated angiogenesis (213). Whether or not the complement system is involved in the latter anti-tumor effects of the anti-cathepsin L treatment remains unresolved.

### Inhibition of Intracellular Protective Pathways

As described above, the list of intracellular molecular pathways supporting cancer cell resistance to CDC is increasing. Currently, we can hypothesize that a coordinated inhibition of any of the following active molecules in the following cancer cells: cAMP, PKC, MEK/ERK, Hsp70, Hsc70, Hsp90, and mortalin, combined with complement-activating antibody, will amplify cancer cell death and increase the sensitivity of cancer to immunotherapy. For each of these molecules, this claim has been clearly supported *in vitro* by data and now awaits *in vivo* testing. Inhibition of PKC and MEK1, the ERK kinase, lowers the rate of MAC elimination from the cells and sensitizes them to CDC (81, 82, 88, 183). Inhibitors of MEK-ERK are in clinical use now in cancer therapy (214–216), and testing their impact on the therapeutic efficacy of anti-cancer antibodies is highly warranted. Heat shock proteins are over-expressed in cancer and play a significant role in resistance to various types of therapy (217). The list of inhibitors of heat shock proteins that have been developed for clinical use is growing and a few have entered clinical trials (90–92, 218, 219). The use of these heat shock protein inhibitors as adjuvants to antibody-based therapy may yield a superior clinical outcome. Mortalin belongs to the family of heat shock proteins and is also over-expressed in cancer (187). The mortalin expression level in colorectal adenocarcinoma cells correlates with poor patient survival (220). Mortalin inhibitors like MKT-077 could be considered as complementary treatment to anticancer antibody therapy. In support, pretreatment with MKT-077 sensitized K562 cells to CDC (87, 189). Unfortunately, thus far, testing of MKT-077 in patients has been stalled due to toxicity effects (221) and alternative inhibitors are being sought. Mortalin silencing with specific siRNA reduced MAC elimination and increased the sensitivity of K562 cells to CDC (189). Therefore, it is reasonable to predict that combining reagents that knockdown or inhibit mortalin with anti-cancer antibody therapy will be advantageous to cancer patients.

## Concluding Remarks

Complement activation on and around cancer cells has been postulated to elicit several concomitant physiological and immunological responses that may act cooperatively to either mediate cancer cell death or promote cell survival, growth, and metastasis. In theory, these responses may also negate and annul each other. Multiple strategies to overcome complement resistance, as described here, open up new opportunities for improving antibody-based immunotherapy. Undoubtedly, applying any of the intervention treatments described above, together with a therapeutic antibody, will produce on and around the cancer cells/mass, besides C5b-9 complexes, additional complement activation products, such as cancer-bound iC3b, which promotes antibody-dependent cellular cytotoxicity (ADCC) and complement-dependent cellular cytotoxicity (CDCC) as well as C3a and C5a, which may suppress cellular anti-cancer immune response. Consequently, in the worst scenario, intervention strategies to augment complement activation may worsen the outcome of the anti-cancer antibody therapy. Hence, for each cancer type, therapeutic antibody, and intervention strategy, an optimal protocol will have to be developed that favors cancer destruction over cancer promotion.

## Author Contributions

All authors listed have made a substantial, direct and intellectual contribution to the work, and approved it for publication.

### Conflict of Interest Statement

The authors declare that the research was conducted in the absence of any commercial or financial relationships that could be construed as a potential conflict of interest.
